# Crystallization and preliminary structure determination of the transfer protein TraM from the Gram-positive conjugative plasmid pIP501

**DOI:** 10.1107/S1744309113000134

**Published:** 2013-01-31

**Authors:** Nikolaus Goessweiner-Mohr, Lukas Grumet, Tea Pavkov-Keller, Ruth Birner-Gruenberger, Elisabeth Grohmann, Walter Keller

**Affiliations:** a Institute for Molecular Biosciences, Karl-Franzens-University Graz, Humboldtstrasse 50/III, 8010 Graz, Styria, Austria; b Institute of Pathology and Center of Medical Research – Core Facility Mass Spectrometry, Medical University Graz, Stiftingtalstrasse 24, 8010 Graz, Styria, Austria; c Division of Infectious Diseases, University Medical Center Freiburg, Hugstetter Str. 55, Freiburg, 79106, Germany

**Keywords:** Gram-positive, conjugative plasmid transfer, pIP501, TraM

## Abstract

The major means of horizontal gene spread (*e.g.* of antibiotic resistance) is conjugative plasmid transfer. It presents a serious threat especially for hospitalized and immuno-suppressed patients, as it can lead to the accelerated spread of bacteria with multiple antibiotic resistances. Detailed information about the process is available only for bacteria of Gram-negative (G−) origin and little is known about the corresponding mechanisms in Gram-positive (G+) bacteria. Here we present the purification, biophysical characterization, crystallization and preliminary structure determination of the TraM C-terminal domain (TraMΔ, comprising residues 190–322 of the full-length protein), a putative transfer protein from the G+ conjugative model plasmid pIP501. The crystals diffracted to 2.5 Å resolution and belonged to space group *P*1, with unit-cell parameters *a* = 39.21, *b* = 54.98, *c* = 93.47 Å, α = 89.91, β = 86.44, γ = 78.63° and six molecules per asymmetric unit. The preliminary structure was solved by selenomethionine single-wavelength anomalous diffraction.

## Abstract 2

This paper reports the successful purification, crystallization and preliminary structure solution of the transfer protein TraM from the Gram-positive conjugative plasmid pIP501.

## 1. Introduction   

Bacterial conjugation is the prevalent means in horizontal gene transfer, by which plasmid-encoded antibiotic resistance and pathogenicity genes are spread (Williams & Hergenrother, 2008 ▶). In the process of conjugation, plasmid DNA is transported from a donor to a recipient cell using a mechanism which requires direct contact between the cells (Cascales & Christie, 2003 ▶; Alvarez-Martinez & Christie, 2009 ▶). A multi-protein complex, large enough to span the bacterial cell wall (Llosa *et al.*, 2002 ▶), handles the transfer. These plasmid-encoded complexes dedicated to the intercellular transport of proteins or protein–DNA complexes are called type IV secretion systems (T4SS). The T4SS have been studied in detail in *Escherichia coli* and *Agrobacterium tumefaciens*, two representatives of Gram-negative bacteria (Llosa *et al.*, 2009 ▶; Hayes *et al.*, 2010 ▶; de La Cruz *et al.*, 2010 ▶; Rêgo *et al.*, 2010 ▶; Smillie *et al.*, 2010 ▶; Wallden *et al.*, 2010 ▶). Most knowledge about Gram-positive T4SS is based on similarity to their Gram-negative counterparts (Grohmann *et al.*, 2003 ▶; Abajy *et al.*, 2007 ▶). However, much more information regarding proteins involved in the T4S processes is available for bacteria of Gram-negative origin (Grohmann *et al.*, 2003 ▶; Kurenbach *et al.*, 2006 ▶; Wallden *et al.*, 2010 ▶; Clewell, 2011 ▶). Only very recently has the first structural information on Gram-positive transfer proteins become available (Porter *et al.*, 2012 ▶; Walldén *et al.*, 2012 ▶).

pIP501, a multiple antibiotic resistance plasmid, was originally isolated from *Streptococcus agalactiae* (Horodniceanu *et al.*, 1979 ▶). It has the broadest known host range for plasmid transfer in Gram-positive bacteria and is furthermore the first conjugative plasmid originating from Gram-positive bacteria for which stable replication in Gram-negative bacteria has been shown (Kurenbach *et al.*, 2003 ▶). Fifteen putative transfer proteins are organized in a single operon, the transfer region. Sequence alignments revealed significant similarity of three pIP501 Tra proteins to the T4SS from *A. tumefaciens*: an ATPase (TraE homologue to VirB4) (Kopec *et al.*, 2005 ▶; Abajy *et al.*, 2007 ▶), a coupling protein (TraJ homologue to VirD4) (Celic *et al.*, unpublished data) and a lytic transglycosylase (TraG homologue to VirB1) (Arends *et al.*, unpublished data). Another member of the pIP501 transfer operon that has been studied in detail is the relaxase TraA (Kopec *et al.*, 2005 ▶; Kurenbach *et al.*, 2006 ▶).

Here we present the purification and crystallization of the deletion mutant protein TraM_190–322_ (formerly called ORF13, GenBank: CAD44393.1; TraM_190–322_ – further referred to as TraMΔ), an 18.6 kDa protein of the T4SS encoded by the conjugative plasmid pIP501. TraMΔ is the first transfer protein of this system to be crystallized. Analytical gel filtration, dynamic light scattering (DLS) and small-angle X-ray scattering (SAXS) show a monomer in solution under the tested conditions. So far, no protein–protein inter­actions of TraM with other pIP501 transfer proteins have been detected (Abajy *et al.*, 2007 ▶) and no relations were found on the sequence level either. As the protein localizes to the cell membrane (Goessweiner-Mohr *et al.*, 2012 ▶), we suggest a role in the scaffolding of the pIP501 core complex.

## 2. Protein purification   

As the full-length protein was insoluble, the putative N-terminal domain and a central *trans*-membrane motif of TraM were deleted and a soluble construct was generated. In brief, *traMΔ* was cloned into the 7×His-tag expression vector pQTEV (a gift from K. Büssow, Max-Planck-Institute for Molecular Genetics, Berlin, Germany) and *E. coli* BL21-CodonPlus (DE3)-RIL (Stratagene, Amsterdam, The Netherlands) competent cells were transformed with the recombinant construct, pQTEV-*traMΔ*. For the selenomethionine expression, pQTEV-*traMΔ* plasmid DNA was isolated and transformed into the methionine-deficient *E. coli* strain B834 (DE3) (Merck, Darmstadt, Germany) using standard protocols.

**Figure 1:**
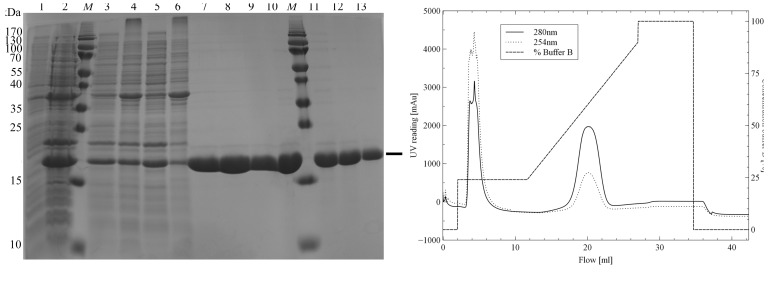
TraMΔ protein production. (*a*) SDS–PAGE to assess protein production and purification (TraMΔ, 18.6 kDa). Lanes 1 and 2, expression before and after 3 h IPTG induction; lanes 3 and 5, supernatant of the two-step extraction; lanes 4 and 6, pellet of the two-step extraction; lanes 7–9, main fractions of the His-affinity purification; lane 10, pooled and concentrated His-affinity fractions; lanes 11–13, main size-exclusion chromatography fractions; lane *M*, molecular-mass marker (PageRuler SM0671, Thermo Fisher Scientific, Waltham, Massachusetts, USA; labelled in kDa). (*b*) His-affinity purification of TraMΔ. The imidazole gradient is shown as the percentage of buffer *B* (discontinued line).

Large-scale expression of TraMΔ was performed in 500 ml LB medium, supplemented with 100 µg ml^−1^ ampicillin. At an OD_600_ of ∼0.6 expression was induced by the addition of 1 m*M* IPTG. After 3 h at 310 K, cells were harvested and immediately frozen at 253 K. TraMΔ expression levels were monitored by SDS–PAGE (Fig. 1 ▶ *a*).

For the expression of the selenomethionine derivative, un-induced cells were harvested at an OD_600_ of ∼0.6, resuspended in M9 minimal medium and growth was continued for an additional hour at 310 K. The cells were induced with 119 mg IPTG, 25 mg of selenomethionine were added and overexpression continued for 3 h. In all preparations, 500 ml of LB media were used. The cells were harvested and immediately frozen at 253 K.

For the purification of the seleno-TraMΔ the cells were resuspended in 40 ml 25 m*M* HEPES pH 7.6, 75 m*M* ammonium sulfate. 2 µl DNAse I (Sigma–Aldrich, St Louis, USA), 1 m*M* phenylmethane­sulfonyl fluoride (PMSF) and 2 m*M* benzamidine were added, the solution was vigorously mixed (UltraTurrax, IKA, Staufen, Germany) and kept on ice for 30 min. The solution was sonicated (Sonopuls HD2070, Bandelin; 1 min, continuous sonification, ∼80% amplitude) and centrifuged for 30 min at 281 K and 15 000*g*. Pellet and supernatant fractions were analysed by SDS–PAGE (Fig. 1 ▶ *a*). The pellet was applied to a second extraction step with 20 ml of the buffer mentioned above, but without additives. TraMΔ-containing supernatants were pooled and loaded onto a HisTrap FF 1 ml column (GE Healthcare, Chalfont St Giles, UK) for affinity purification (Fig. 1 ▶ *b*). The purity of TraMΔ was assessed by SDS–PAGE (Fig. 1 ▶ *a*). Imidazole was removed by buffer exchange during concentrating (Amicon tubes, 3000 MWCO, Merck Millipore, Darmstadt, Germany).

**Figure 2:**
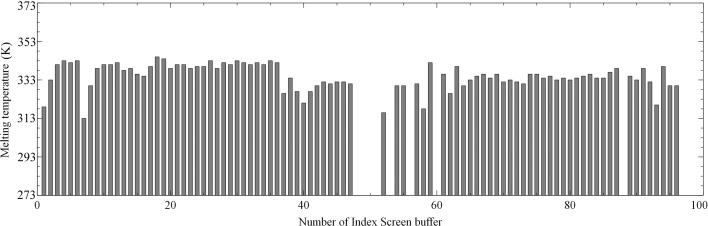
Example of the buffer-optimization assays. The melting temperatures (K) of TraMΔ are plotted as a function of the buffer and differ significantly corresponding to the respective chemical composition. The values on the *x* axis correspond to the numbering of the Index crystallization screen. Missing values represent melting curves that were measured but were not interpretable, probably due to precipitation or aggregation of the protein.

Purified TraMΔ protein with a concentration of 1 mg ml^−1^ was applied to an adapted Thermofluor buffer optimization screen (Ericsson *et al.*, 2006 ▶) using the conditions of various commercial crystallization screens: Index and Crystal Screen and Crystal Screen 2 (Hampton Research, Aliso Viejo, California, USA), as well as Morpheus and JCSG (Molecular Dimensions, Newmarket, Suffolk, UK). For the screen, 10 µl of protein sample were mixed with 10 µl of the respective buffer and 5 µl of 50× SYPRO Orange (Sigma–Aldrich, St Louis, USA). The resulting thermostability curves were analysed (see Fig. 2 ▶ as an example), an optimized extraction buffer was designed, combining the buffer components (Collins *et al.*, 2004 ▶) which showed a thermostabilizing effect, while keeping the composition as simple as possible. This buffer consisted of 50 m*M* HEPES pH 7.0, 200 m*M* ammonium sulfate and was used for all subsequent TraMΔ extractions, as well as for crystallization.

## 3. Biophysical characterization   

**Figure 3:**
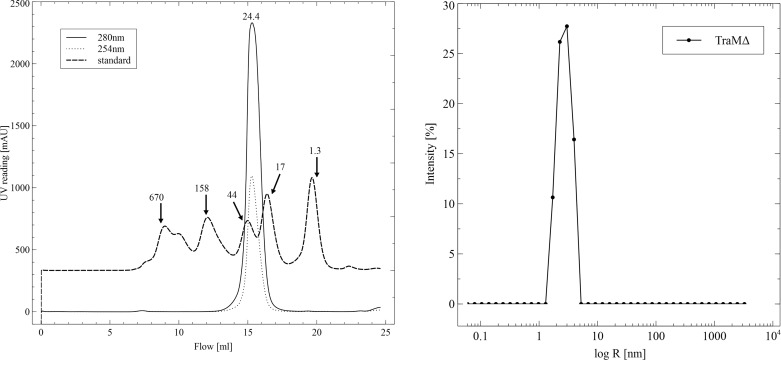
Biophysical characterization of TraMΔ. (*a*) TraMΔ elutes as a single peak from the Superdex 200 size-exclusion column. The 280 nm (solid line) and 254 nm (dotted line) readings are shown. A standard (BioRad) is shown with its molecular weight (discontinued). (*b*) In the monodispersity analysis *via* DLS TraMΔ appears as a narrow peak.

For the biophysical characterization, TraMΔ was extracted and His-affinity purified in 50 m*M* Tris pH 7.45, 200 m*M* ammonium sulfate. TraMΔ-containing His-affinity fractions were pooled and concentrated to a concentration of 2.2 mg ml^−1^ *via* centrifugation in Amicon tubes (3000 MWCO). TraMΔ was further purified by size-exclusion chromatography with a Superdex 200 HR 10/30 column (GE Healthcare). A gel-filtration standard (BioRad, Hercules California, USA; 670/158/44/17/1.35 kDa) was used to calculate the molecular weight of TraMΔ. TraMΔ eluted from the gel-filtration column as a single peak (Fig. 3 ▶ *a*), indicative of a homogeneous species with an apparent molecular weight of 24.4 kDa. This value compares to the theoretical molecular weight of the His-tagged construct of 18.6 kDa, suggesting that TraMΔ is a monomer in solution.

The mono-dispersity of TraMΔ was evaluated by DLS. For the DLS measurements, a size-exclusion fraction, containing 0.9 mg ml^−1^ TraMΔ, was measured directly in a 45 µl cuvette. Ten measurements with constant baseline were merged, yielding a single peak with a calculated polydispersity of 26.7% and a hydrodynamic (*R*_h_) radius of 2.8 nm (Fig. 3 ▶ *c*).

**Figure 4:**
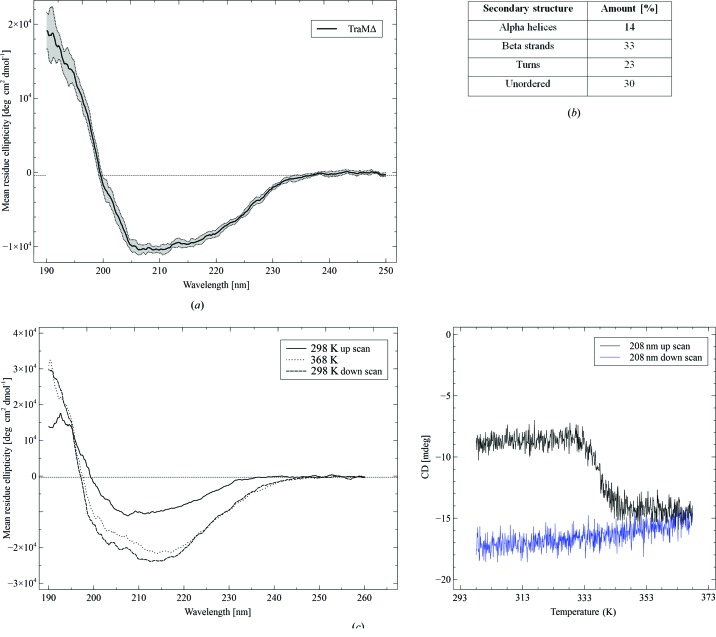
CD analysis of TraMΔ. (*a*) TraMΔ is folded in solution. The black curve represents the average of ten individual wavelength scans. The standard deviation is displayed as a shaded area. (*b*) Secondary structure content of TraMΔ. The NRMSD (normalized root mean square deviation) is 0.018. (*c*) TraMΔ unfolding and refolding characteristics. The CD spectra are shown at 298 and 368 K and after cooling to 298 K (left panel). The temperature scan at 208 nm (up- and down-scan) is shown in the right panel.

Circular dichroism (CD) measurements were performed on a Jasco J715 (JASCO Inst., Gross-Umstadt, Germany) spectro-polarimeter equipped with an external thermostat. Spectra were measured from 260 to 190 nm in a 0.01 cm cuvette and with a protein concentration of 0.9 mg ml^−1^. Ten individual spectra were accumulated and the standard deviation was calculated from the repeated measurements. Temperature scans were performed in a 0.02 cm temperature-controlled cuvette in the range from 298 to 368 K using a step-scan procedure with a constant wavelength of 208 nm. Spectra resulted from three accumulated scans, which were measured from 260 to 190 nm every 5 K. The temperature gradient was set to 1 K min^−1^. TraMΔ was applied at a concentration of 0.45 mg ml^−1^. The CD data were evaluated using the online service Dichroweb (Whitmore & Wallace, 2008 ▶) with reference database No. 4. Purified TraMΔ is folded in solution and has a mixed α–β composition (Fig. 4 ▶ *a*). The amount of β-sheets exceeds that of α-helices by more than two times (Fig. 4 ▶ *b*). The large proportion of unordered structure (30%) may result from flexible N- or C-terminal parts. Temperature scans revealed that TraMΔ undergoes a transition at 338 K (Fig. 4 ▶ *c*), but does not unfold completely even at 368 K. Instead the CD spectrum at 368 K shows the characteristics of a protein with increased β-sheet contents. As the protein is trapped in this state (*i.e.* no refolding during the down-scan), we call the state of TraMΔ upon heating ‘β-arrest’.

SAXS measurements were performed to gain more information about the oligomeric state and shape of TraMΔ in solution. For the measurements on the X33 beamline (DESY, Hamburg, Germany), TraMΔ was suspended in 100 m*M* ammonium sulfate, 100 m*M* NaCl, 50 m*M* HEPES pH 7.0. Size-exclusion purified protein was concentrated to a final concentration of 2.8 mg ml^−1^. TraMΔ was measured at three different concentrations: 2.65/1.25/0.65 mg ml^−1^. The program *PRIMUS* (Konarev *et al.*, 2003 ▶) was used for data analysis, yielding an *I*_0_ of 19.46, a radius of gyration (*R*_g_) of 2.5 nm and a *D*_max_ of 8 nm, as calculated from the Guinier plot (data at 1.25 mg ml^−1^) and the *p*(*r*) function, respectively. The radius of gyration is in good agreement with the hydrodynamic radius (2.8 nm) determined by DLS measurements. From *I*_0_ we calculated the apparent molecular weight of TraMΔ in solution, using BSA (bovine serum albumin) as a molecular-weight standard (Pavkov *et al.*, 2008 ▶). The value of 20.1 kDa is in good agreement with the theoretical molecular weight of TraMΔ (18.6 kDa) and with the observation from gel filtration (24.4 kDa). Calculating *ab initio* models from the scattering function, we observed an elongated particle, which may be due to the flexible N-terminal end of TraMΔ, containing the unstructured 7×His tag.

## 4. Crystallization   

All crystallization experiments were performed with an Oryx8 robot (Douglas Instruments, East Garston, Hungerford, Berkshire, UK) using the microbatch method (Chayen *et al.*, 1992 ▶). The following screens were used: Index, Crystal Screen and Crystal Screen 2, PEG/Ion (Hampton Research) and JCSG, Morpheus (Molecular Dimensions). The protein concentrations used were between 4 and 6 mg ml^−1^ and the drop ratio was 1:1 with a total drop volume of 1 µl. All plates were covered with paraffin oil (∼4 ml) and stored at 293 K. Protein crystals were tested for diffraction on a rotating-anode diffractometer (MicroStar, Bruker AXS, Madison, Wisconsin, USA). The only positive candidate condition [Index No. 44: 0.1 *M* HEPES pH 7.5, 25%(*w*/*v*) PEG 3350] was used for microbatch pH/PEG optimization with constant protein drop ratios of 35 and 50%(*v*/*v*). The protein concentration was lowered further, to facilitate slower crystal growth. Since there are no protein structures with significant sequence similarity to TraMΔ available, molecular replacement was not an option for structure solution. Thus, all optimizations were performed with the selenomethionine derivative of TraMΔ, leading to the final conditions: protein stock 3.0 mg ml^−1^; drop volume 2 µl (0.7 µl protein solution, 1.3 µl precipitant solution); 0.1 *M* HEPES pH 7.33, PEG 3350 16.5%(*v*/*v*).

**Figure 5:**
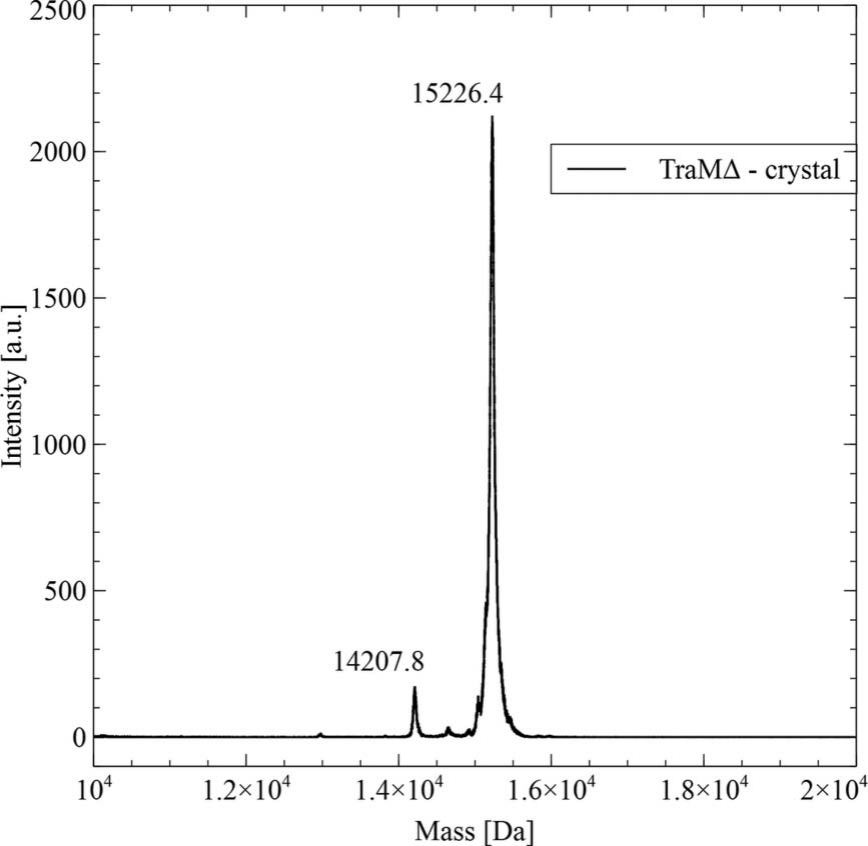
MALDI–TOF analysis of TraMΔ crystals.

To confirm the integrity of TraMΔ in the crystals, we analysed dissolved crystals *via* mass spectroscopy (MS). Several crystals of TraMΔ were dissolved in 10 µl of pure H_2_O and investigated by matrix-assisted laser desorption/ionization–time-of-flight (MALDI–TOF) analysis (Bruker, ultrafleXtreme, Vienna, Austria). This experiment showed that the protein present in the crystals (Fig. 5 ▶; 15.2 kDa) was significantly smaller than the original His-tagged construct (18.6 kDa), with the 3.4 kDa difference representing approximately 30 residues. These residues were lost due to unintended *in situ* proteolytic activity during the crystallization. Subsequently, one of the samples was digested with trypsin and further analysed *via* MS/MS, yielding the N-terminal sequence ‘SVKKESEL’ and a sequence coverage of 130 residues (193 to 322 of the original TraM sequence), resulting in a theoretical molecular mass of 15232 Da.

## 5. Data collection and processing   

**Figure 6:**
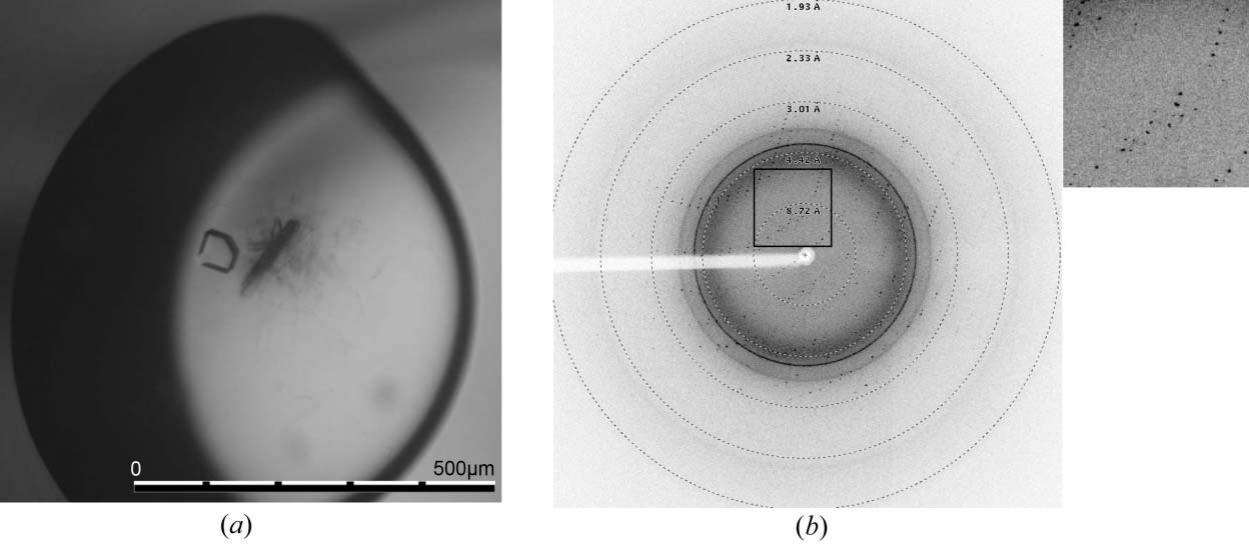
TraMΔ crystallization and data collection. (*a*) A representative TraMΔ crystal, with compact growth with a size of less than 100 µm. The crystal was grown using the microbatch method at 293 K and with paraffin oil for sealing the plate. The protein drop ratio was 35% with a protein stock concentration of 3.0 mg ml^−1^. The drop size was 2 µl with the following final conditions derived from Index condition No. 44: 16.5% PEG 3350, 0.1 *M* HEPES, pH 7.33. (*b*) Diffraction pattern of a TraMΔ selenomethionine crystal obtained using synchrotron radiation on beamline X06DA, SLS, Villigen, Switzerland; resolution rings have been added. The picture was generated using ADXV (A. Arvail). Inset, detail of the diffraction shown in (*b*).

Crystals were flash-cooled without cryoprotectant (Fig. 6 ▶ *a*). Data collection was performed at 100 K on the synchrotron beamline X06DA at SLS, Villigen, Switzerland. Most of the tested selenomethionine-containing crystals diffracted to a limited resolution (weak spots to less than 7 Å resolution in the best direction) on our home source and at the synchrotron. A single crystal showed diffraction with clear spots and a non-twinned pattern at better than 2.5 Å resolution at the SLS (Fig. 6 ▶ *b*). A fluorescence scan was performed to validate the presence of selenomethionine in the crystal and to define the optimal setup for anomalous data collection at the seleno *f*′′ peak wavelength (0.9792 Å). A crystal-to-detector distance of 200 mm, an oscillation range of 1.0° and an exposure time of 1 s per image were chosen. Three individual data sets were collected at different spots on the same crystal and later scaled together.

The crystals belonged to space group *P*1, with unit-cell parameters *a* = 39.21, *b* = 54.98, *c* = 93.47 Å, α = 89.91, β = 86.44, γ = 78.63° and six molecules per asymmetric unit. The Matthews coefficient (Matthews, 1968 ▶; Kantardjieff & Rupp, 2003 ▶) was calculated as 2.16 Å^3^ Da^−1^, with a solvent content of 43.04% (Tables 1 ▶ and 2 ▶). In order to determine the internal symmetry we performed a self-rotation function (Tollin & Rossmann, 1966 ▶) yielding a strong threefold axis and three perpendicular twofold axes, which indicates six molecules in the asymmetric unit.

The data sets were processed and scaled together using the programs *XDS* and *XSCALE* (Kabsch, 2010 ▶). The programs *AutoSol* (McCoy *et al.*, 2007 ▶; Terwilliger *et al.*, 2009 ▶) and *AutoBuild* (Terwilliger *et al.*, 2008 ▶) of the *PHENIX* software suite (Adams *et al.*, 2010 ▶) were used to define the seleno heavy-atom sites and to build a first model. The generated electron-density map, including the Hendrickson–Lattman coefficients and heavy-atom coordinates, was put in *BUCCANEER* (Cowtan, 2006 ▶). The resulting model was completed manually and is currently being refined. The final TraMΔ structure has been published (Goessweiner-Mohr *et al.*, 2012 ▶).

## Figures and Tables

**Table 1:** Data-collection and processing statistics of scaled data Values in parentheses are for the highest-resolution shell.

Beamline	X06DA (PXIII), SLS, Villigen, Switzerland
Space group	*P*1
Detector	MAR CCD
Unit-cell dimensions (, )	*a* = 39.21, *b* = 54.98, *c* = 93.47, = 89.91, = 86.44, = 78.63
Wavelength ()	0.9792
Resolution range ()	502.5 (2.62.5)
*R*_meas_ † (%)	12.8 (53.1)
*I*/(*I*)	14.91 (4.05)
No. of molecules in asymmetric unit	6
Matthews coefficient (^3^Da^1^)	2.16
Solvent content (%)	43.04
Unique reflections	51575 (5801)
Measured reflections	293455 (31927)
Redundancy	5.7 (5.5)
Completeness (%)	97.5 (97.7)

† *R*_meas_ = 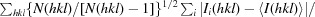 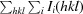.

**Table 2:** Results for the Matthews coefficient calculation Values calculated for a molecular weight of 15226Da. Nmol/asym = no. of molecules in asymmetric unit.

Nmol/asym	Matthews coefficient	Solvent (%)	Probability (*N*) for given resolution (2.5)	Probability (*N*) overall
1	12.95	90.51	0	0
2	6.47	81.01	0	0
3	4.32	71.52	0.01	0.01
4	3.24	62.03	0.10	0.11
5	2.59	52.53	0.40	0.39
6	2.16	43.04	0.43	0.43
7	1.85	33.55	0.05	0.05
8	1.62	24.05	0	0
9	1.44	14.56	0	0
10	1.29	5.07	0	0

## References

[bb1] Abajy, M. Y., Kopeć, J., Schiwon, K., Burzynski, M., Döring, M., Bohn, C. & Grohmann, E. (2007). *J. Bacteriol.* **189**, 2487–2496. 10.1128/JB.01491-06 PMC1899387 17209024

[bb2] Adams, P. D. *et al.* (2010). *Acta Cryst.* D**66**, 213–221.

[bb3] Alvarez-Martinez, C. E. & Christie, P. J. (2009). *Microbiol. Mol. Biol. Rev.* **73**, 775–808. 10.1128/MMBR.00023-09 PMC2786583 19946141

[bb4] Cascales, E. & Christie, P. J. (2003). *Nature Rev. Microbiol.* **1**, 137–149. 10.1038/nrmicro753 PMC3873781 15035043

[bb5] Chayen, N. E., Shaw Stewart, P. D. & Blow, D. M. (1992). *J. Cryst. Growth*, **122**, 176–180.

[bb6] Clewell, D. B. (2011). *Mob. Genet. Elements*, **1**, 38–54. 10.4161/mge.1.1.15409 PMC3190283 22016844

[bb7] Collins, B. K., Tomanicek, S. J., Lyamicheva, N., Kaiser, M. W. & Mueser, T. C. (2004). *Acta Cryst.* D**60**, 1674–1678. 10.1107/S090744490401844X 15333952

[bb8] Cowtan, K. (2006). *Acta Cryst.* D**62**, 1002–1011. 10.1107/S0907444906022116 16929101

[bb9] de la Cruz, F., Frost, L. S., Meyer, R. J. & Zechner, E. L. (2010). *FEMS Microbiol. Rev.* **34**, 18–40. 10.1111/j.1574-6976.2009.00195.x 19919603

[bb10] Ericsson, U. B., Hallberg, B. M., Detitta, G. T., Dekker, N. & Nordlund, P. (2006). *Anal. Biochem.* **357**, 289–298. 10.1016/j.ab.2006.07.027 16962548

[bb11] Goessweiner-Mohr, N., Grumet, L., Arends, K., Pavkov-Keller, T., Gruber, C. C., Gruber, K., Birner-Gruenberger, R., Kropec-Huebner, A., Huebner, J., Grohmann, E. & Keller, W. (2012). *J. Biol. Chem.* **288**, 2018–2028. 10.1074/jbc.M112.428847 PMC3548508 23188825

[bb12] Grohmann, E., Muth, G. & Espinosa, M. (2003). *Microbiol. Mol. Biol. Rev.* **67**, 277–301. 10.1128/MMBR.67.2.277-301.2003 PMC156469 12794193

[bb13] Hayes, C. S., Aoki, S. K. & Low, D. A. (2010). *Annu. Rev. Genet.* **44**, 71–90. 10.1146/annurev.genet.42.110807.091449 21047256

[bb14] Horodniceanu, T., Bougueleret, L., El-Solh, N., Bouanchaud, D. H. & Chabbert, Y. A. (1979). *Plasmid*, **2**, 197–206. 10.1016/0147-619x(79)90038-6 109871

[bb15] Kabsch, W. (2010). *Acta Cryst.* D**66**, 125–132. 10.1107/S0907444909047337 PMC2815665 20124692

[bb16] Kantardjieff, K. A. & Rupp, B. (2003). *Protein Sci.* **12**, 1865–1871. 10.1110/ps.0350503 PMC2323984 12930986

[bb17] Konarev, P. V., Volkov, V. V., Sokolova, A. V., Koch, M. H. J. & Svergun, D. I. (2003). *J. Appl. Cryst.* **36**, 1277–1282.

[bb18] Kopec, J., Bergmann, A., Fritz, G., Grohmann, E. & Keller, W. (2005). *Biochem. J.* **387**, 401–409. 10.1042/BJ20041178 PMC1134968 15554903

[bb19] Kurenbach, B., Bohn, C., Prabhu, J., Abudukerim, M., Szewzyk, U. & Grohmann, E. (2003). *Plasmid*, **50**, 86–93. 10.1016/s0147-619x(03)00044-1 12826062

[bb20] Kurenbach, B., Kopeć, J., Mägdefrau, M., Andreas, K., Keller, W., Bohn, C., Abajy, M. Y. & Grohmann, E. (2006). *Microbiology*, **152**, 637–645. 10.1099/mic.0.28468-0 16514144

[bb21] Llosa, M., Gomis-Rüth, F. X., Collect, M. & de la Cruz Fd, F. (2002). *Mol. Microbiol.* **45**, 1–8. 10.1046/j.1365-2958.2002.03014.x 12100543

[bb22] Llosa, M., Roy, C. & Dehio, C. (2009). *Mol. Microbiol.* **73**, 141–151. 10.1111/j.1365-2958.2009.06751.x PMC2784931 19508287

[bb23] McCoy, A. J., Grosse-Kunstleve, R. W., Adams, P. D., Winn, M. D., Storoni, L. C. & Read, R. J. (2007). *J. Appl. Cryst.* **40**, 658–674. 10.1107/S0021889807021206 PMC2483472 19461840

[bb35] Matthews, B. W. (1968). *J. Mol. Biol.* **33**, 491–497. 10.1016/0022-2836(68)90205-2 5700707

[bb24] Pavkov, T., Egelseer, E. M., Tesarz, M., Svergun, D. I., Sleytr, U. B. & Keller, W. (2008). *Structure*, **16**, 1226–1237. 10.1016/j.str.2008.05.012 18682224

[bb25] Porter, C. J., Bantwal, R., Bannam, T. L., Rosado, C. J., Pearce, M. C., Adams, V., Lyras, D., Whisstock, J. C. & Rood, J. I. (2012). *Mol. Microbiol.* **83**, 275–288. 10.1111/j.1365-2958.2011.07930.x 22150951

[bb26] Rêgo, A. T., Chandran, V. & Waksman, G. (2010). *Biochem. J.* **425**, 475–488. 10.1042/BJ20091518 20070257

[bb27] Smillie, C., Garcillán-Barcia, M. P., Francia, M. V., Rocha, E. P. & de la Cruz, F. (2010). *Microbiol. Mol. Biol. Rev.* **74**, 434–452. 10.1128/MMBR.00020-10 PMC2937521 20805406

[bb28] Terwilliger, T. C., Adams, P. D., Read, R. J., McCoy, A. J., Moriarty, N. W., Grosse-Kunstleve, R. W., Afonine, P. V., Zwart, P. H. & Hung, L.-W. (2009). *Acta Cryst.* D**65**, 582–601. 10.1107/S0907444909012098 PMC2685735 19465773

[bb29] Terwilliger, T. C., Grosse-Kunstleve, R. W., Afonine, P. V., Moriarty, N. W., Zwart, P. H., Hung, L.-W., Read, R. J. & Adams, P. D. (2008). *Acta Cryst.* D**64**, 61–69. 10.1107/S090744490705024X PMC2394820 18094468

[bb30] Tollin, P. & Rossmann, M. G. (1966). *Acta Cryst.* **21**, 872–876. 10.1107/s0365110x66004109 6013452

[bb31] Wallden, K., Rivera-Calzada, A. & Waksman, G. (2010). *Cell. Microbiol.* **12**, 1203–1212. 10.1111/j.1462-5822.2010.01499.x PMC3070162 20642798

[bb32] Walldén, K., Williams, R., Yan, J., Lian, P. W., Wang, L., Thalassinos, K., Orlova, E. V. & Waksman, G. (2012). *Proc. Natl Acad. Sci. USA*, **109**, 11348–11353. 10.1073/pnas.1201428109 PMC3396474 22745169

[bb33] Whitmore, L. & Wallace, B. A. (2008). *Biopolymers*, **89**, 392–400. 10.1002/bip.20853 17896349

[bb34] Williams, J. J. & Hergenrother, P. J. (2008). *Curr. Opin. Chem. Biol.* **12**, 389–399. 10.1016/j.cbpa.2008.06.015 PMC2570263 18625335

